# Angiotensin AT2 Receptor is Anti-inflammatory and Reno-Protective in Lipopolysaccharide Mice Model: Role of IL-10

**DOI:** 10.3389/fphar.2021.600163

**Published:** 2021-04-15

**Authors:** Naureen Fatima, Sanket Patel, Tahir Hussain

**Affiliations:** Department of Pharmacological and Pharmaceutical Sciences, College of Pharmacy, University of Houston, Houston, TX, United States

**Keywords:** Lipopolysaccharide, angiotensin II type 2 receptor, interleukin-10, neutralizing IL-10 antibody, pro-inflammatory cytokines, kidney injury

## Abstract

Acute kidney injury (AKI) due to endotoxemic insult is predicted by the infiltration of neutrophils, monocytes and macrophages, and the release of pro-and anti-inflammatory cytokines to the site of injury. Earlier, we have demonstrated the role of angiotensin-II type 2 receptor (AT2R) stimulation in reno-protection in lipopolysaccharide (LPS)-induced inflammation and AKI in C57BL6/NHsd mice. Moreover, AT2R activation has been shown to increase the anti-inflammatory cytokine interleukin-10 (IL-10), its role in AT2R-mediated anti-inflammation and reno-protection is unknown. To address this question, in the present study mice were treated with the AT2R agonist C21 (0.3 mg/kg, intraperitoneally), LPS (5 mg/kg, intraperitoneally), or LPS with C21 pre-treatment with or without neutralizing IL-10 antibody. Treatment with C21 alone caused an increase in the plasma and kidney IL-10 levels, which peaks at 2-h, and returned to baseline at 6-h. The C21-induced IL-10 increase was blocked by the AT2R antagonist PD123319 suggesting AT2R’s involvement. LPS treatment caused a profound increase in tumor necrosis factor-α (TNF-α) and interleukin-6 (IL-6) and the LPS-induced increase in these cytokines was attenuated by the C21 pre-treatment (1-h prior LPS) both in the plasma and kidney. Neutralizing IL-10 antibody treatment abrogated the C21-lowering of TNF-α and IL-6 in the kidney but not in the plasma. Similar results as related to the cytokines profiles in all the groups were also observed in the heart and spleen. The alteration in early cytokine profile prompted us to measure the markers of renal function (blood urea nitogen and urinary creatinine) in order to analyze the effect of IL-10 neutralization. However, it was too early to observe changes in renal function. Therefore, the renal function and injury markers were again measured at 24 h. Treatment with neutralizing IL-10 antibody attenuated the C21-mediated improvement in indices of the kidney function, but not the biomarkers of renal injury (kidney injury molecule-1 and neutrophil-gelatinase associated lipocalin). Collectively, our data suggest that the involvement of IL-10 in AT2R-mediated anti-inflammation and reno-protection against LPS is complex, mediating the renal cytokine profile and kidney filtration function, but not the plasma cytokine profile and renal injury markers.

## Introduction

The renin-angiotensin system (RAS) is a critical hormonal system, which regulates a balance between its two receptors namely angiotensin-II (ang-II) type 1 (AT1R) and type 2 receptor (AT2R) to mitigate renal injury and inflammation. The ang-II/AT1R axis has been extensively studied for its role in promoting renal inflammation, however, the role of AT2R has become increasingly evident as anti-inflammatory in renal and cardiovascular diseases (Sabuhi et al., 2011; Dhande et al., 2013; Skorska et al., 2015; Patel et al., 2016; Lange et al., 2018; Patel et al., 2019). The hallmarks of early renal inflammation include the release of cytokines and recruitment of neutrophils, monocytes, and macrophages to the site of injury (Rabb et al., 2016). Stimulation of AT2R inhibits early renal inflammation in renovascular hypertension (Matavelli et al., 2011) and diabetes (Matavelli et al., 2015). AT2R stimulation by its agonist C21 exerts anti-inflammatory role in human renal proximal tubular epithelial cells (HK-2 cells), THP-1 macrophages as well as in animal models of acute kidney injury (AKI) and chronic kidney diseases (Dhande et al., 2013; Dhande et al., 2015; Patel et al., 2016; Patel et al., 2019).

Lipopolysaccharide (LPS), a Toll-like receptor 4 (TLR4) ligand, activates nuclear factor-kappa-β (NF-ĸβ) and stimulates the production of pro-inflammatory cytokines TNF-α and IL-6 (Lawrence, 2009). LPS challenge is commonly used to elucidate the mechanism of AKI following a strong immune response and local infiltration of immune cells. It generates a cytokine storm in the early phase even before the onset of AKI (Bihorac et al., 2013). Dhande et al. reported that AT2R activation caused a decrease in TNF-α and IL-6, and an increase in IL-10 in LPS activated HK-2 cells and in the kidney of obese Zucker rats (Dhande et al., 2013). Furthermore, prior activation of AT2R with C21 attenuated the LPS-induced increase in pro-inflammatory cytokine production in THP-1 macrophages, via a sustained increase in ERK1/2 phosphorylation (Dhande et al., 2015). Moreover, AT2R stimulation by C21 enhanced IL-10 production in HK-2 cells without an inflammatory stimulus as opposed to activation of THP-1 cells by LPS required for IL-10 release. Recent findings from our lab indicate that prior activation of AT2R by C21 rather than concurrent activation was anti-inflammatory against LPS-induced inflammation and AKI in mice (Patel et al., 2019). Interestingly, an earlier study has shown that pre-treatment with IL-10 prevents LPS toxicity by reducing the release of TNF-α (Gerard et al., 1993). However, the role of IL-10 in AT2R-mediated anti-inflammatory and reno-protective responses against LPS-induced kidney injury is unknown. Therefore, the present study was aimed to elucidate the involvement of IL-10 in the AT2R-mediated anti-inflammatory and reno-protective effect against LPS challenge.

## Methods

### Animals

Male C57BL/6NHsd mice (8–10 weeks of age) were purchased from Harlan Laboratories (Madison, WI), and were housed and acclimatized in the University of Houston animal care facility for 1 week. Animals were kept on a 12 h/12 h light/dark cycle at temperature 71–72°F with free access to standard chow and tap water. The experimental protocol was approved by the University of Houston Institutional Animal Care and Use Committee.

### Treatment Protocols

All drug administrations were intraperitoneal (n = 3–16). C21, PD123319 and LPS were dissolved in normal saline. The volumes of the drugs were calculated based on the concentration of the drug and body weight of mice. The concentration of C21 was 0.3 mg/5 ml, PD123319 was 5 mg/ml and LPS was 1 mg/ml.

#### Protocol 1

Mice were randomly divided into 5 groups: i) Control (normal saline) (n = 16), ii) C21-1h (n = 6), iii) C21-2h (n = 6), iv) C21-6h (n = 3) and v) C21–24 h (n = 3); equi-volume of normal saline or a single dose of C21 (0.3 mg/kg, i.p.) was administered to each mouse of respective group, and they were euthanized after 1, 2, 6 and 24 h, respectively. C21 is a kind gift from Vicore Pharma, Sweden.

#### Protocol 2

Mice were randomly divided into 4 groups: i) Control (normal saline) (n = 10), ii) C21 (n = 6), iii) PD (n = 5) and iv) PD + C21 (n = 5). AT2R antagonist PD123319 (16099, Cayman chemicals) was administered (5 mg/kg, i.p.) 15 min prior to a single dose of C21 (0.3 mg/kg, i. p.). All mice were euthanized 2-h after C21 treatment.

#### Protocol 3

Mice were randomized into 7 groups (n = 6 each): i) Control (isotype), ii) C21, iii) LPS, iv) C21 + LPS, v) Neutralizing IL-10 antibody (IL-10Ab)+C21, vi) IL-10Ab + C21 + LPS vii) IL-10Ab + LPS. LPS (L2880, Sigma Aldrich) was given at a dose of 5 mg/kg. Rat IgG1 kappa isotype control (eBRG1, Thermo Fisher Scientific) and a neutralizing IL-10 monoclonal antibody (JES5-2A5, Thermo Fisher Scientific) were given a dose of 200 μg/mice in a total volume of 200 μL. IL-10 Ab treatment was given 1-h prior to C21, and LPS treatment was given 1-h after C21. Mice were euthanized 1-h post LPS.

#### Protocol 4

Mice were randomized to seven groups (n = 5 each) as per Protocol 3. After LPS treatment, they were housed in the metabolic cages for collection of 24-h urine. Mice were euthanized 24-h post LPS treatment.

Table 1 represents Protocol 3 and 4 in a more simplified manner.

**TABLE 1:** Summary of treatments at different time points (Protocol 3 and 4).

Time (hour)	0 h	1 h	2 h	1 h/24 h post LPS
Control	Normal saline	Isotype control	Normal saline	Euthanize
C21	Normal saline	C21	Normal saline	
LPS	Normal saline	Normal saline	LPS	
C21 + LPS	Normal saline	C21	LPS	
IL-10Ab + C21	IL-10Ab	C21	Normal saline	
IL-10Ab + C21 + LPS	IL-10Ab	C21	LPS	
IL-10Ab + LPS	Normal saline	IL-10Ab	LPS	

All mice were euthanized under isoflurane anesthesia. Spot urine was collected from all mice in Protocol 1–3. Twenty 4-h urine was collected from mice in Protocol 4. Blood for plasma was collected via cardiac puncture into Vaccutainer^®^ K3-EDTA-coated tubes. Plasma was separated by centrifugation at 700 × g for 20 min at 4°C. Tissues including kidney, heart, and spleen were collected. Tissue samples and aliquots of urine and plasma samples were stored at −80°C for further analysis.

### Tissue Homogenate Preparation

Tissues (∼10–15 mg) were homogenized (10% w/v) by the mechanical disruption in ice-cold lysis buffer (150 mM sodium chloride, 1 mM ethylenediamine tetraacetic acid, 1 mM ethylene glycol-bis(β-aminoethyl ether)-N,N,N′,N′-tetraacetic acid, 1% TritonX-100, 0.5% sodium deoxycholate, 1% protease-phosphatase inhibitor cocktail, all dissolved in 1X Tris buffer) followed by centrifugation at 700 × g for 30 min at 4°C. The pellets were discarded and the supernatants containing tissue homogenates were stored in aliquots at −80°C.

### IL-10 Neutralization

The bioactivity of IL-10 was blocked in the plasma and kidney homogenates by treatment with neutralizing IL-10 monoclonal antibody (JES5-2A5). This was done to ensure that there was no free IL-10 in the plasma or kidney homogenates. Control mice were treated with Rat IgG1 kappa isotype control (eBRG1).

### Cytokine Measurements by ELISA

The cytokines IL-10, TNF-α, and IL-6 were quantified in the plasma and tissue homogenates of mice (in protocol 1–3) using Mouse Quantikine ELISA kits M1000B, MTA00B and M6000B, respectively (R&D Systems Inc.) as per manufacturer’s instructions.

### Measurement of Kidney Injury Markers

Plasma and urinary biomarkers of renal function such as blood urea nitrogen (BUN) (QuantiChrome urea assay kit, DIUR-100), and creatinine (QuantiChrome creatinine assay kit, DICT-500) were measured spectrophotometrically according to manufacturer’s instructions (BioAssay Systems). The kidney injury was assessed (in protocol 4) by detecting KIM-1 and NGAL in the kidney homogenates using ELISA kits MKM100 and MLCN20, respectively (R&D Systems Inc.).

### Statistical Analysis

Data are presented as mean ± S.E.M. One-way Analysis of Variance (ANOVA) with Fisher’s LSD test for multiple comparisons was used to compare variations between more than two groups. A value of *p* < 0.05 was considered statistically significant, n = 3–16 (protocol 1), n = 5–10 (protocol 2), n = 6 (protocol 3) and n = 5 (protocol 4). A total of 121 mice were used in this study.

## Results

### Time Course of IL-10 Release by C21 Induced AT2R Stimulation

**FIGURE 1:**
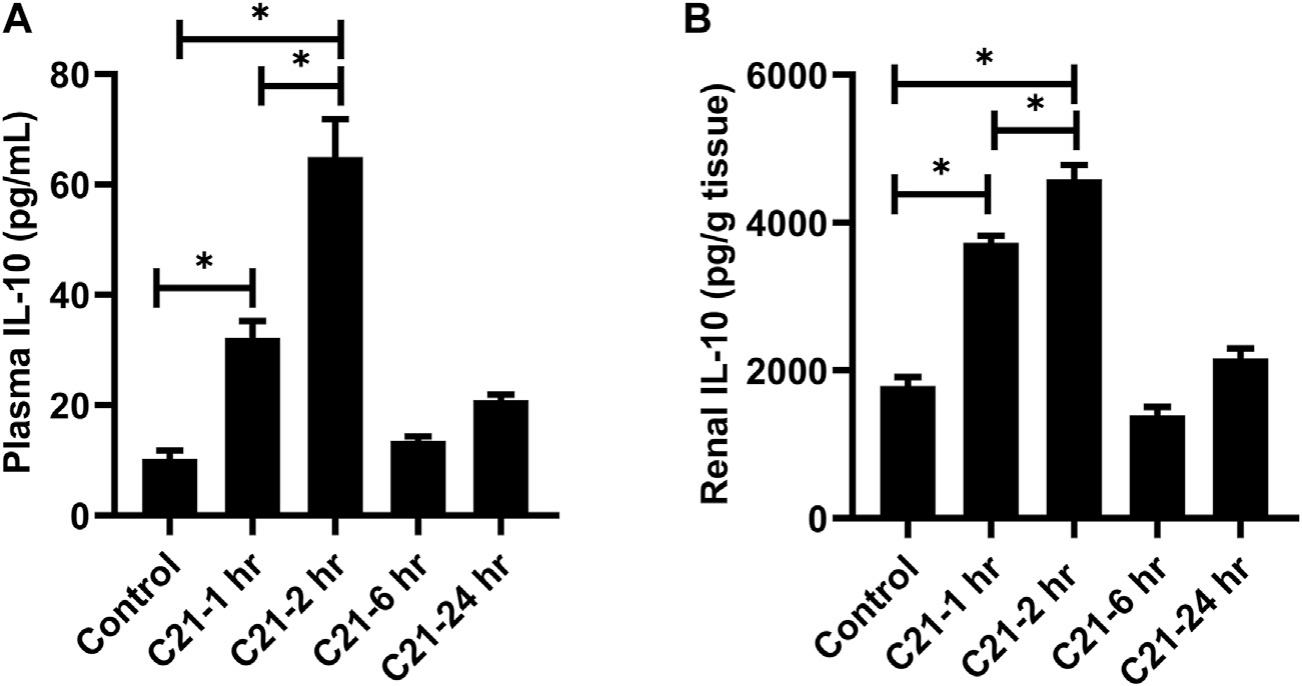
Time course for the release of IL-10 followed by C21 treatment in the **(A)** plasma and **(B)** kidney of C57BL6/NHsd mice. Data are represented as Mean ± S.E.M., analyzed by one-way ANOVA with Fisher’s LSD test for multiple comparisons and are considered significant at *p < 0.05.

Figures 1A,B represent the time profile of IL-10 released when AT2R is stimulated by C21 for 1-, 2-, 6-, and 24-h in the plasma and kidney. It was found that IL-10 levels increase in both plasma and kidney at 1-h post C21, and peak around 2-h, but then begin to decline. It returns to almost normal levels similar to control by 24 h.

### Release of IL-10 is AT2R Specific

**FIGURE 2:**
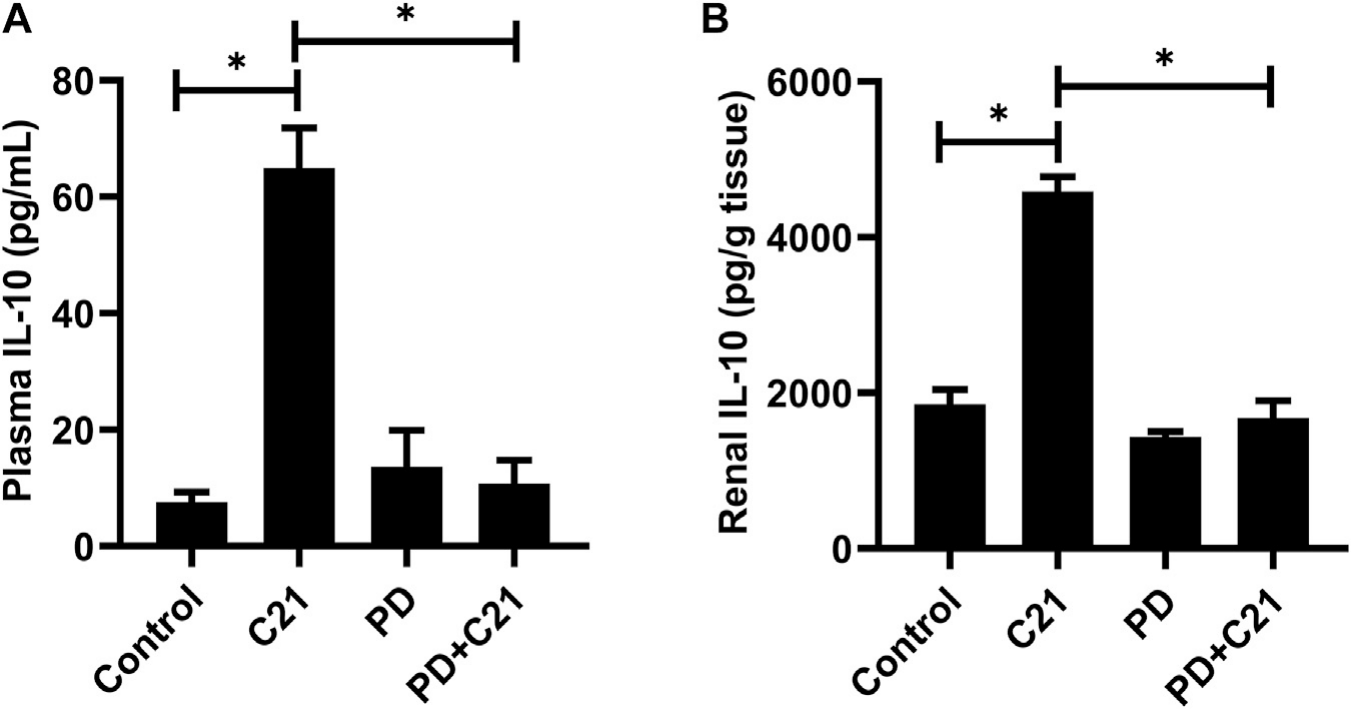
Inhibition of the anti-inflammatory effect of C21 by AT2R antagonist PD in the **(A)** plasma and **(B)** kidney of C57BL6/NHsd mice. Data are represented as Mean ± S.E.M., analyzed by one-way ANOVA with Fisher’s LSD test for multiple comparisons and are considered significant at *p < 0.05.

Treatment with C21 led to an increase in IL-10 levels, which was significantly attenuated by treatment with the AT2R antagonist PD in both plasma and kidney, suggesting the involvement of AT2R (Figures 2A,B).

### Measurement of Cytokines by ELISA

**FIGURE 3:**
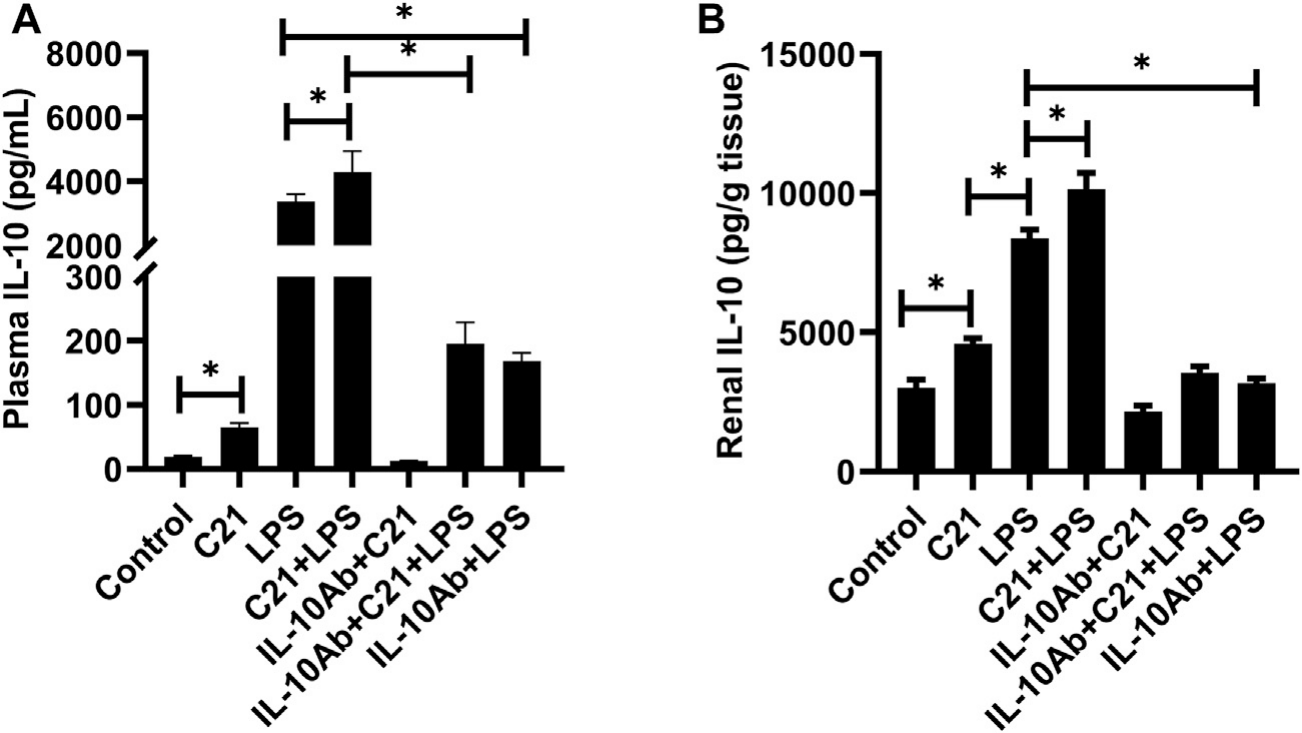
IL-10 concentration in the **(A)** plasma and **(B)** kidney of C57BL6/NHsd mice treated with C21, LPS, or both with and without neutralizing IL-10 antibody. Data are represented as Mean ± S.E.M., analyzed by one-way ANOVA with Fisher’s LSD test for multiple comparisons and are considered significant at *p < 0.05.

Figures 3A,B represent the effect of different treatment combinations on the plasma and kidney levels of anti-inflammatory cytokine IL-10. C21 treatment increased the IL-10 levels significantly than control (isotype) mice in both plasma and kidney. The LPS challenge induced significantly higher levels of IL-10, which were further increased by C21 pre-treatment. Furthermore, treatment with neutralizing IL-10 antibody significantly decreased the IL-10 release in either C21, LPS, or C21 pretreated LPS samples with the same pattern followed in both plasma and kidney.

**FIGURE 4:**
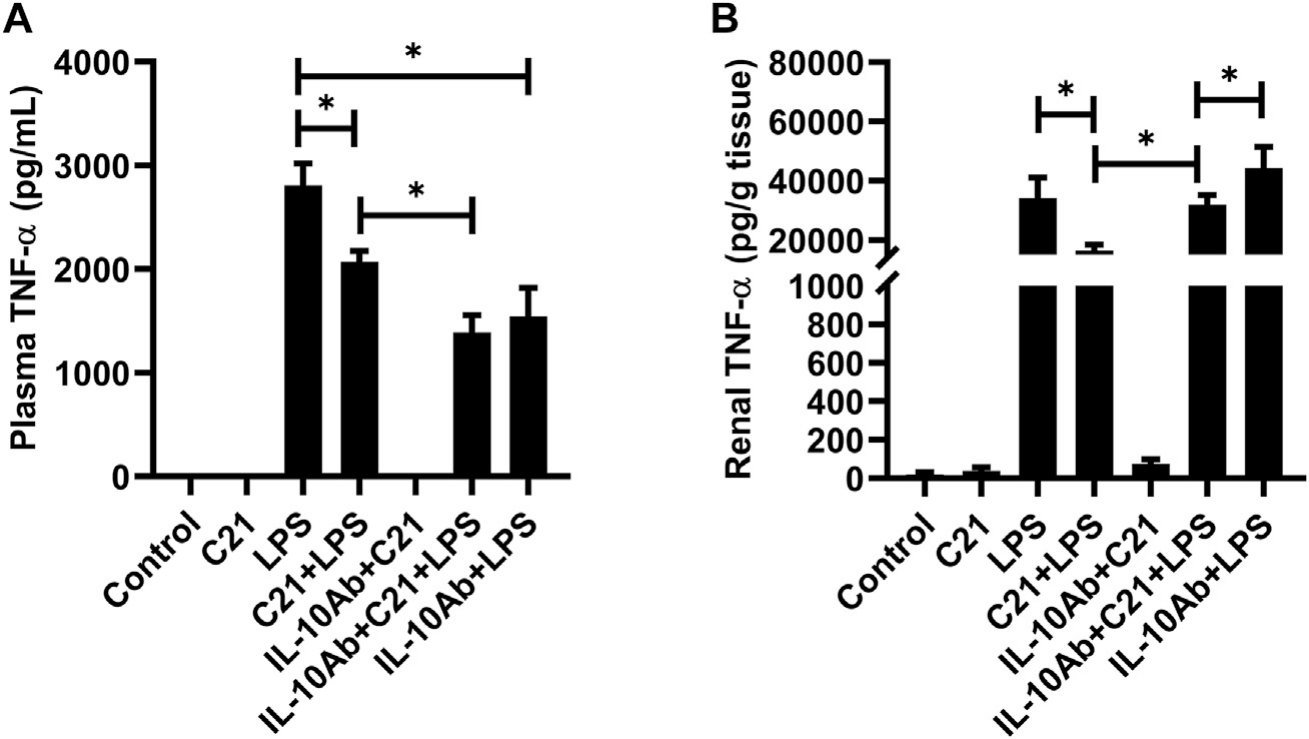
TNF-α concentration in the **(A)** plasma and **(B)** kidney of C57BL6/NHsd mice treated with C21, LPS, or both with and without neutralizing IL-10 antibody. Data are represented as Mean ± S.E.M., analyzed by one-way ANOVA with Fisher’s LSD test for multiple comparisons and are considered significant at *p < 0.05.

Figures 4A,B represent the plasma and kidney levels of pro-inflammatory cytokine TNF-α on treatment with C21, or LPS, or both either with or without the neutralizing IL-10 antibody treatment. TNF-α was found in very low concentrations in the plasma and kidney homogenates from isotype control, C21 and IL-10Ab + C21 groups. LPS induced very high levels of TNF-α in the plasma and kidney, which were significantly lowered by C21 treatment. Upon IL-10 neutralization, C21 mediated decrease in TNF-α bounced back to the LPS level in the kidney, but not in the plasma. In fact, the plasma levels of TNF-α were further decreased after IL-10 neutralization. When IL-10 was neutralized in LPS alone mice, plasma TNF-α did not significantly change, but in the kidney, the levels were further significantly higher as compared to the IL-10Ab + C21 + LPS group. In plasma, the IL-10Ab + LPS group had significantly lower TNF-α as compared to the LPS alone group. In the kidney, there was no significant difference between the TNF-α levels between the two groups.

**FIGURE 5:**
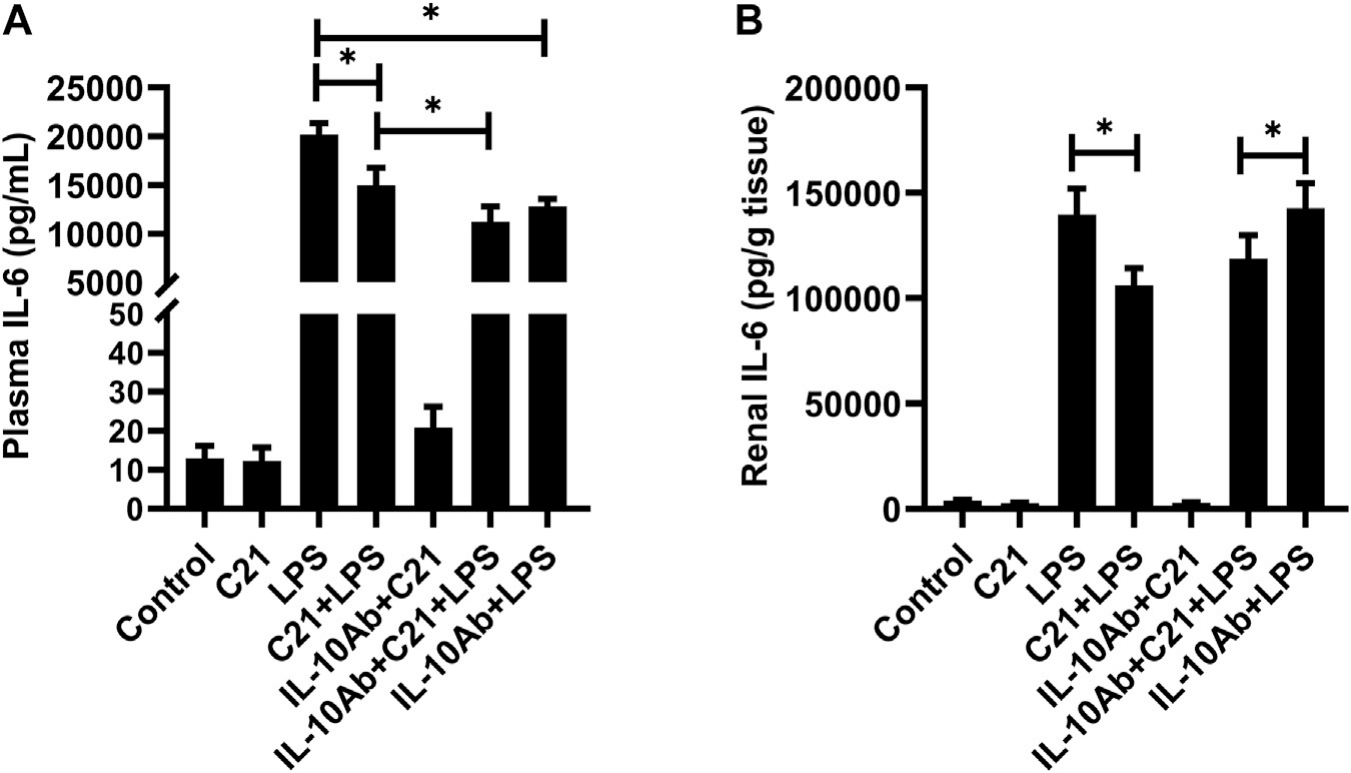
IL-6 concentration in the **(A)** plasma and **(B)** kidney of C57BL6/NHsd mice treated with C21, LPS, or both with and without neutralizing IL-10 antibody. Data are represented as Mean ± S.E.M., analyzed by one-way ANOVA with Fisher’s LSD test for multiple comparisons and are considered significant at *p < 0.05.

Figures 5A,B represent the plasma and kidney IL-6 levels on treatment with C21, LPS, or LPS pre-treated with C21 groups, and the effect of neutralizing IL-10 antibody treatment upon the inflammatory status in these groups. It was found that IL-6 followed a similar pattern as of the TNF-α levels. LPS induced tremendously high levels of plasma and kidney IL-6, but C21 was able to modestly but significantly lower IL-6 levels in both plasma and kidney. Treatment with neutralizing IL-10 antibody attenuated the C21-mediated decrease in IL-6 levels in the kidney, but not in the plasma. IL-6 levels tend to increase again in IL-10Ab + C21 + LPS group, but this increase was non-significant as compared to C21 + LPS group, in contrast to kidney TNF-α levels which was significantly higher as compared to C21 + LPS group. In IL-10Ab + LPS group, IL-6 levels do not change in the plasma but increase slightly (but significantly) in the kidney as compared to IL-10Ab + C21 + LPS group. The difference in IL-6 levels between LPS and neutralizing IL-10Ab + LPS groups was statistically significant in the plasma but not in the kidney.

### Plasma and Urinary Biomarkers of Kidney Injury

**FIGURE 6:**
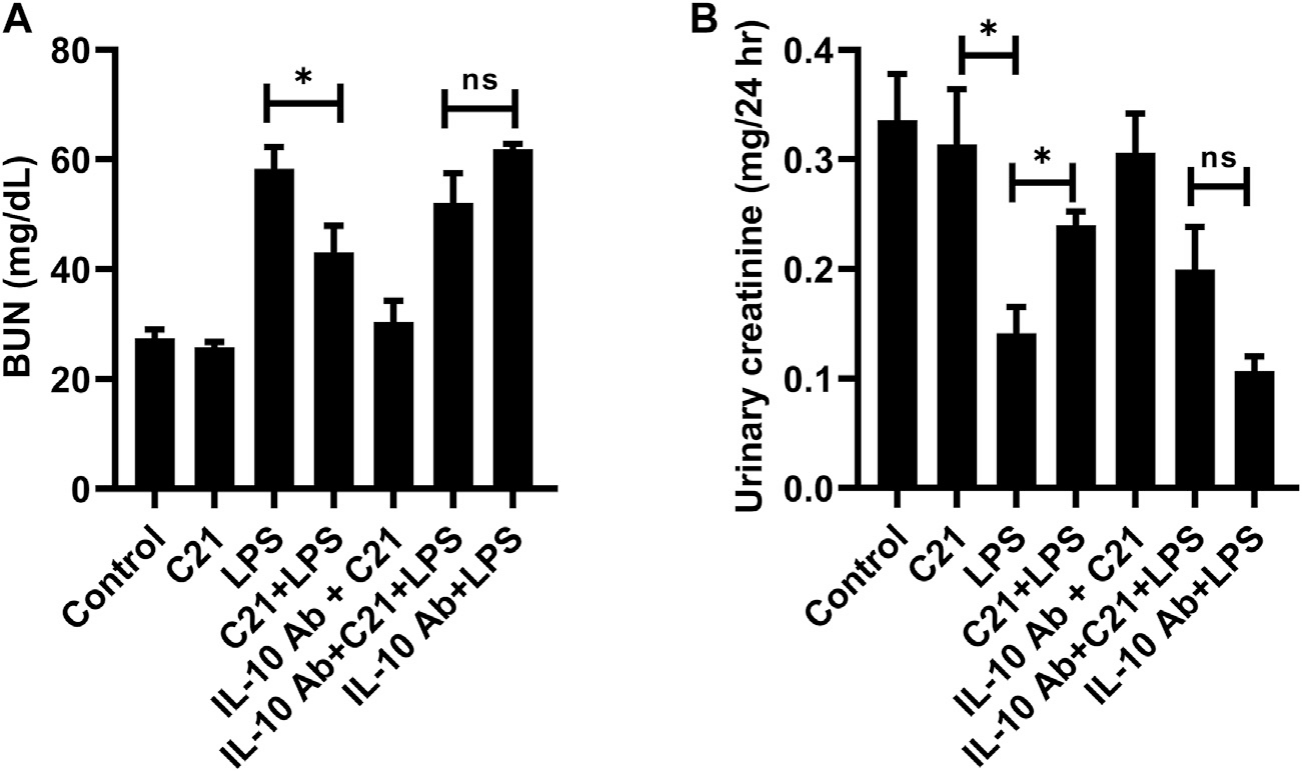
Measurement of functional indices of the kidney **(A)** BUN and **(B)** urinary creatinine following 24 h LPS challenge in C57BL6/NHsd mice treated with C21, LPS, or both with and without neutralizing IL-10 antibody. Data are represented as Mean ± S.E.M., analyzed by one-way ANOVA with Fisher’s LSD test for multiple comparisons and are considered significant at *p < 0.05.

Kidney filtration function was assessed by measuring BUN and urinary creatinine in mice (of protocol 4). Figure 6A represents the level of BUN which increased significantly in LPS treated mice as compared to isotype control mice. This increase was significantly reversed by C21 prior treatment. IL-10 neutralization tends to reverse the C21-mediated decrease in BUN levels, although this increase was not statistically significant. Furthermore, the urinary creatinine levels were also measured in order to analyze the effect of IL-10 neutralization on functional injury to the kidney (Figure 6B). Treatment with LPS for 24 h significantly decreased (>50%) the normal levels of urinary creatinine, but 1-h prior treatment with C21 markedly improved the urinary creatinine levels. This improvement in the urinary creatinine levels was shown to decline again upon IL-10 neutralization, which was worsened in the IL-10Ab + LPS group. However, there was no significant difference in the BUN and urinary creatinine levels between IL-10Ab + C21 + LPS and IL-10Ab + LPS groups either.

**FIGURE 7:**
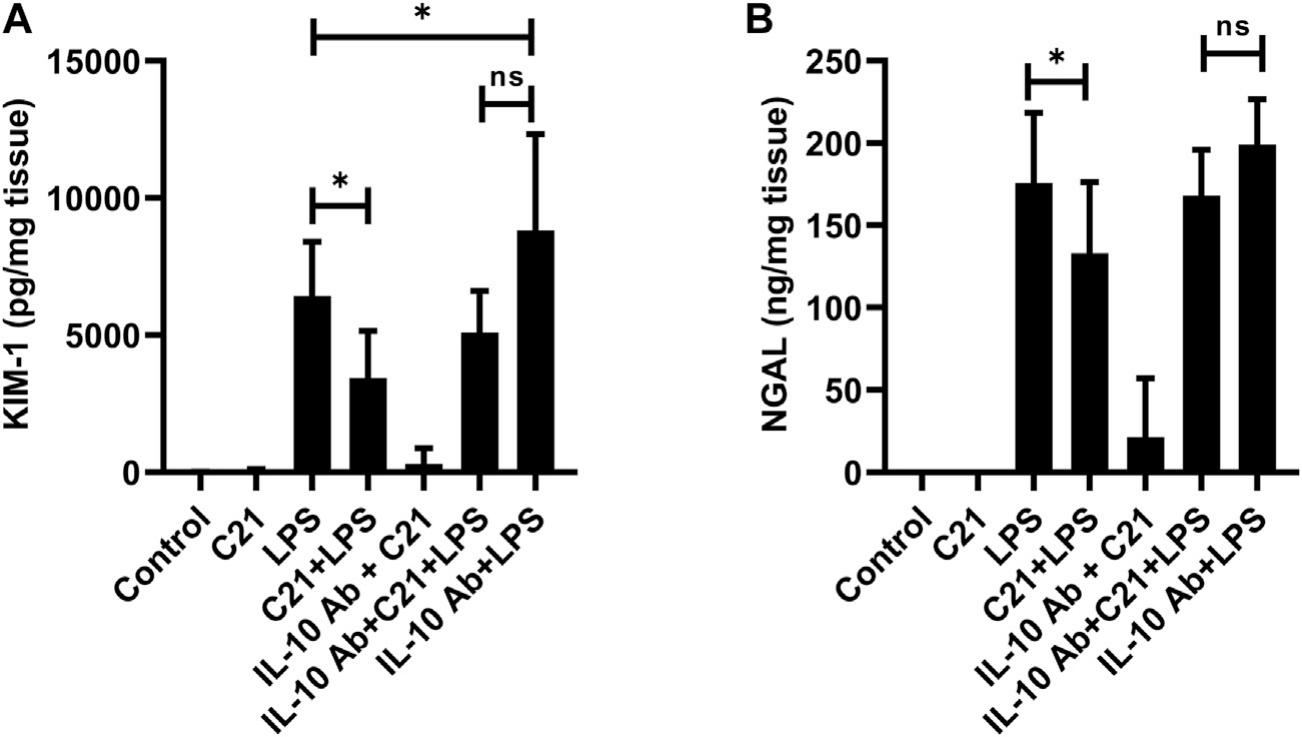
Measurement of renal tubular injury markers **(A)** KIM-1 and **(B)** NGAL following 24 h LPS challenge in C57BL6/NHsd mice treated with C21, LPS, or both with and without neutralizing IL-10 antibody. Data are represented as Mean ± S.E.M., analyzed by one-way ANOVA with Fisher’s LSD test for multiple comparisons and are considered significant at *p < 0.05.

Besides the indices of kidney function, the biomarkers of kidney injury KIM-1 and NGAL were also measured in the kidney tissue homogenates (Figures 7A,B). The levels of KIM-1 and NGAL were not detectable in isotype control, C21 or IL-10Ab + C21 groups. LPS treatment tremendously increased the levels of these markers as compared to controls. C21 prior treatment significantly brought down these levels. However, IL-10 neutralization did not significantly attenuate the protective effect of C21 against LPS-induced increase in these injury markers. When the changes in the levels of KIM-1 and NGAL between LPS and C21 + LPS were compared with the changes between IL-10Ab + LPS and IL-10Ab + C21 + LPS, it was found that C21 induced almost the same amount of change in both the cases, regardless of whether IL-10Ab was present or not.

## Discussion

Endotoxin-induced AKI due to LPS is characterized by a systemic response of overwhelming pro-inflammatory cytokines and recruitment of inflammatory cells at the site of injury (Doi et al., 2009). An immense increase in the levels of inflammatory mediators immediately following LPS challenge is an early predictor of developing acute kidney injury (Zhang et al., 2015). In this study, it was aimed to elucidate the role of IL-10 in the anti-inflammatory and reno-protective effect of acute AT2R stimulation against LPS challenge. We have used a single dose of C21 for stimulation of AT2R for studying the beneficial effects of C21 at early (1-h post-LPS) and later (24-h post-LPS) time points.

Our results clearly demonstrate that AT2R stimulation by C21, without any inflammatory stimulus, increased the plasma and renal IL-10 levels within 1-h with a peak at around 2-h, and then start to decline. By 24-h, the level returns to normal, as seen in control mice. The level of IL-10 was at its peak at 2-h in the heart and spleen tissue as well (Supplementary Figure S1). Also in human kidney HK-2 proximal tubule epithelial cells, C21 alone stimulated the production of IL-10 without LPS treatment (Dhande et al., 2013). However, in THP-1 cells (macrophages), C21 treatment alone did not stimulate IL-10 production without an inflammatory stimulus, such as LPS (Dhande et al., 2015), suggesting a complex nature between AT2R and IL-10 production in immune vs non-immune cells. The involvement of AT2R in the release of IL-10 was confirmed by the use of AT2R antagonist PD123319, which decreased the levels of IL-10 in the plasma, kidney, heart and spleen (Supplementary Figure S2) which is in line with other studies in kidney cortex and THP-1 cells from our laboratory (Dhande et al., 2013; Dhande et al., 2015).

The use of neutralizing antibody is a viable approach to elucidate the beneficial role of a protein of interest after blocking its function, as seen in a study where IL-10 reversed the Dinitrobenzene-sulfonic acid-induced colitis in mice, and this anti-inflammatory effect was blocked by treatment with neutralizing IL-10 antibody (Hunter et al., 2005). The freely available IL-10 was significantly blunted (almost 50%) within 1-h of treatment with a single dose (200 μg/mice) of neutralizing IL-10 antibody in both plasma and kidney of C21 treated mice (Supplementary Figure S3). Furthermore, since C21 induced IL-10 release which peaked at 2-h, LPS treatment was given 1-h after C21 so that IL-10 production remained in progress before the inflammatory stimulus came into action.

LPS drives the release of high amounts of IL-10 both in the plasma and kidney, as also observed in other studies (Patel et al., 2019; Dhande et al., 2015; Pengal et al., 2006; Iyer et al., 2010; Lobo-Silva et al., 2016). C21 further increased the IL-10 levels significantly as seen in our previous studies. It was observed that C21 alone did not induce the release of any of these pro-inflammatory cytokines, but LPS alone led to the release of a significant amount of these cytokines in the plasma and kidney. Interestingly, prior treatment with C21 modestly but significantly decreased the plasma and kidney levels of TNF-α and IL-6. Perhaps, AT2R-mediated activation of phosphatases could be a mechanism in reducing the phosphorylation and subsequent activation of NF-ĸB, a master regulator involved in formation of cytokines. The reduced expression of TLR4 due to stimulation of AT2R during an LPS challenge was also observed in mice (unpublished data). Furthermore, the interesting observation in this study was that on treatment with IL-10 neutralizing antibody, these pro-inflammatory cytokines further decreased in the plasma and bounced back to the LPS level in the kidney. This indicates that C21 is anti-inflammatory against LPS induced inflammation via IL-10 in the kidney but not in the plasma. There seems to be a different mechanism of protection by C21 in the plasma under the same treatment conditions. Both TNF-α and IL-6 followed the same pattern in plasma or kidney. In addition, in the IL-10Ab + LPS group, the level of both the cytokines in the kidney was even higher when compared to the IL-10Ab + C21 + LPS group, further indicating that C21 is protecting against LPS-induced renal inflammation. This increase was not as prominent in the plasma for both the cytokines, probably because protection mediated by C21 is not via IL-10. Another important observation here is that the plasma concentration of these pro-inflammatory molecules (TNF-α/IL-6) is ∼10-times less as compared to that in the kidney which might indicate that the renal cells and renal AT2R as relevant and important player in inflammation and injury. Perhaps, plasma is not an appropriate compartment to judge the anti-inflammatory effect of AT2R.

Besides, the insult caused to the kidney by LPS may have recruited more than one type of infiltrating pro-inflammatory cells, including macrophages as shown by numerous studies (Shahin et al., 1987; Rampanelli et al., 2013; Yu et al., 2015; Mir et al., 2018; Nakano et al., 2020). Macrophages express a local independently functional RAS within them (Nataraj et al., 1999; Okamura et al., 1999; Jurewicz et al., 2007). Additionally, resident macrophages are also present in the kidney (Munro and Hughes, 2017). Our work and previous literature has suggested the involvement of proximal tubular epithelial cells (Dhande et al., 2013), macrophages (Dhande et al., 2015), T-cells (Curato et al., 2010; Skorska et al., 2015), dendritic cells (Ma et al., 2019) and Treg cells (Ali et al., 2021) as a source of IL-10 upon AT2R stimulation. This could be responsible for higher concentrations of renal IL-10 as compared to plasma. Moreover, the presence of cytokines in the plasma and their activity do not necessarily go side-by-side. Although high plasma levels may reflect that the production of cytokines is high but their levels do not necessarily mean that their bioactivity is enhanced (Tetta et al., 2003). This might indicate that the existing IL-10 levels in the plasma were not sufficient for its bioactivity needed for C21 to display anti-inflammation. The presence or absence of cytokines in biological fluids depends on many factors including activating and inhibitory signals, production and breakdown, and binding to the target cell (Cavaillon et al., 1992). Moreover, the metabolism of any protein or receptor is quite different in tissues than in plasma (Torell et al., 2015). Besides, the local RAS is independently regulated and compartmentalized from the plasma circulation (Velez, 2009). This may indicate that the receptors of RAS could be acting via a different mechanism in different compartments that needs to be investigated.

**FIGURE 8:**
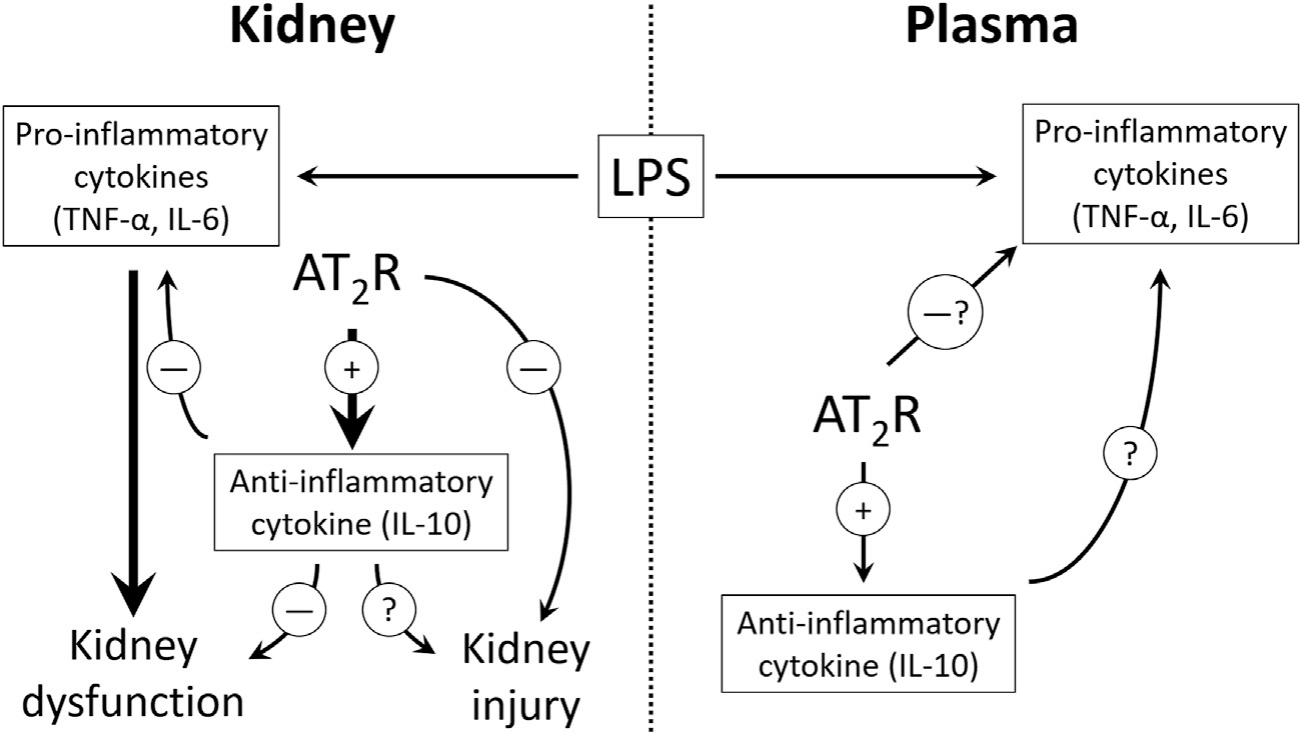
The proposed potential mechanism of AT2R mediated anti-inflammation and reno-protection. 10. Table legend.

The alteration in early cytokine profile following 1-h LPS challenge, and the worsening of renal inflammation upon IL-10 neutralization, indicating IL-10 involvement in AT2R mediated anti-inflammation in the kidney, prompted us to think if IL-10 neutralization could have any effect on kidney filtration function and/or injury. In order to predict functional changes in the kidney, BUN and urinary creatinine levels were measured in samples from protocol 3. It was found that BUN values were slightly increased and urinary creatinine levels were slightly decreased after LPS treatment, and C21 showed protection against these deviations (data not shown). However, upon IL-10 neutralization, C21 still seemed protective, but since the animals were euthanized just after 1-h, this effect did not seem very definite, and indicated that 1-h might not be enough to bring about any significant kidney dysfunction or injury. Therefore, another set of animals were treated with the same regimen and euthanized 24 h after LPS treatment (Protocol 4). Renal functional indices BUN and creatinine, and tubular injury are early diagnostic markers for AKI (Basile et al., 2012; Fattah and Vallon, 2018) and the protective role of AT2R against IR-induced AKI has been demonstrated in another study in our laboratory (Ali et al., 2021). In view of the findings from our laboratory in I/R and LPS models of AKI, the levels of BUN and urinary creatinine were again measured in the 24-h samples. These functional indices of the kidney were significantly altered due to LPS treatment, and 1-h prior treatment with C21 was significantly protective against LPS-induced increased BUN and decreased urinary creatinine levels, which is consistent with our previous study (Patel et al., 2019). IL-10 neutralization clearly reduced the protective effect of C21, by increasing BUN, and decreasing urinary creatinine levels closer to LPS group, which is consistent with the renal cytokine profile. However, since these changes were not significantly different from C21 + LPS group, we could only say that there is a partial involvement of IL-10 in attenuating the C21-mediated protection against kidney dysfunction. Furthermore, since the filtration function of kidney was greatly impacted 24 h post LPS, it was reasonable to study the markers of kidney injury. Therefore, KIM-1 and NGAL were measured, which are biomarkers for early diagnosis of AKI, and increased upon damage to the normal kidney morphology, especially tubular injury involving the proximal tubular epithelial cells (Vaidya et al., 2008; Simsek et al., 2013; Luo et al., 2016). The level of these markers were significantly high in the kidney of 24-h LPS treated animals, which is consistent with other studies (Bondeva et al., 2018; Kim et al., 2020). C21 reduced the LPS-driven increase in KIM-1 and NGAL in the kidney homogenates, although these changes were not very dramatic, but still statistically significant as compared to LPS. However, if it is observed carefully, the amount of decrease in the level of these markers by C21 was either same or even more after neutralizing IL-10, which indicates that IL-10 might not be involved in C21-mediated improvement in the markers of kidney injury, rather only partially involved in improving the kidney function. Therefore, it is clear from the findings of this study that IL-10 neutralization prevented AT2R-mediated protection in terms of renal cytokine profile and kidney filtration function, but not plasma cytokines and kidney injury (Figure 8). As mentioned earlier, the differential response to IL-10 neutralization in plasma and kidney could also be a result of heterogeneous cell populations in the kidney and circulation. Furthermore, it is also noteworthy that although there was almost 50% neutralization of the IL-10 antibody, but still it was an incomplete neutralization, the reason for which remains unknown at this point. This could be another reason for a partial involvement of IL-10 in the anti-inflammatory and reno-protective response of AT2R against LPS challenge.

Therefore, it may be concluded from this study that acute stimulation of AT2R by agonist C21 is anti-inflammatory and reno-protective against LPS. This beneficial effect of AT2R activation could be a result of partial involvement of IL-10. However, the complexity of the results from this study involving the neutralization of IL-10 is difficult to comprehend which needs to be explored further to have a clear insight into the role of AT2R in reno-protection, particularly against LPS-induced inflammation and AKI.

## Data Availability

The raw data supporting the conclusions of this article will be made available by the authors, without undue reservation.

## Abbreviations

AKI, acute kidney injury; ang-II, angiotensin-II; AT1R, angiotensin-II type 1 receptor; AT2R, angiotensin-II type 2 receptor; LPS, lipopolysaccharide; IL-10, interleukin-10; RAS, renin-angiotensin system; TLR4, Toll-like receptor 4; NF-ĸβ, nuclear factor-kappa-β, TNF-α, tumor necrosis factor-α; IL-6, interleukin-6; BUN, blood urea nitrogen; KIM-1, kidney injury molecule-1; NGAL, neutrophil-gelatinase associated lipocalin.
