# Supplementary material for: Angiotensin AT2 Receptor is Anti-inflammatory and Reno-Protective in Lipopolysaccharide Mice Model: Role of IL-10
Source: Front Pharmacol. 2021 Apr 15;12:600163. doi: 10.3389/fphar.2021.600163 (PMC8082177; doi:10.3389/fphar.2021.600163)
Supplement: Supplementary file 1 [file datasheet1.docx]

**Supplementary figures**


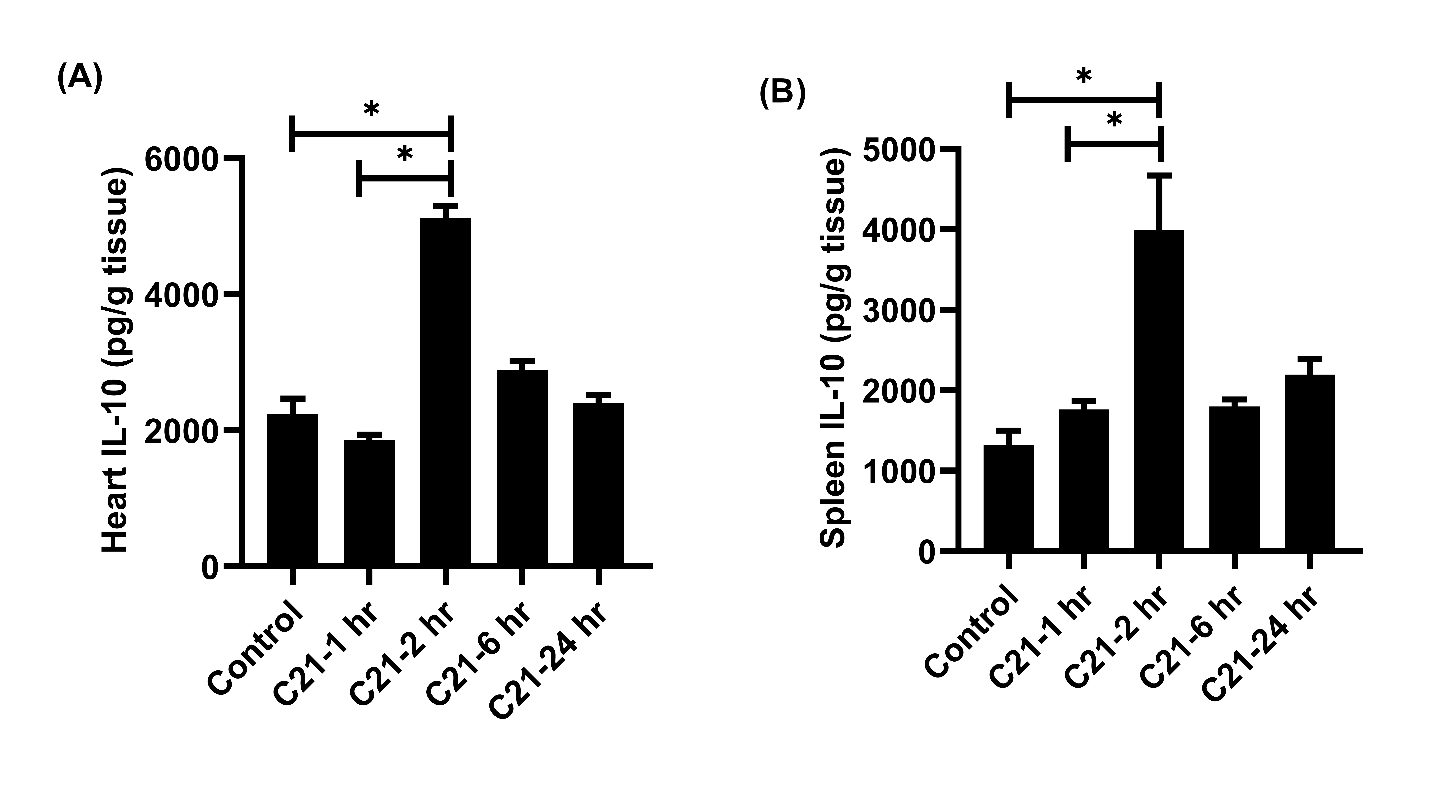


**Supplementary Figure 1:** Time course for the release of IL-10 followed by C21 treatment in the **(A)** heart and **(B)** spleen of C57BL6/NHsd mice. Data are represented as Mean ± S.E.M., analyzed by one-way ANOVA with Fisher’s LSD test for multiple comparisons and are considered significant at **P*<0.05.

**
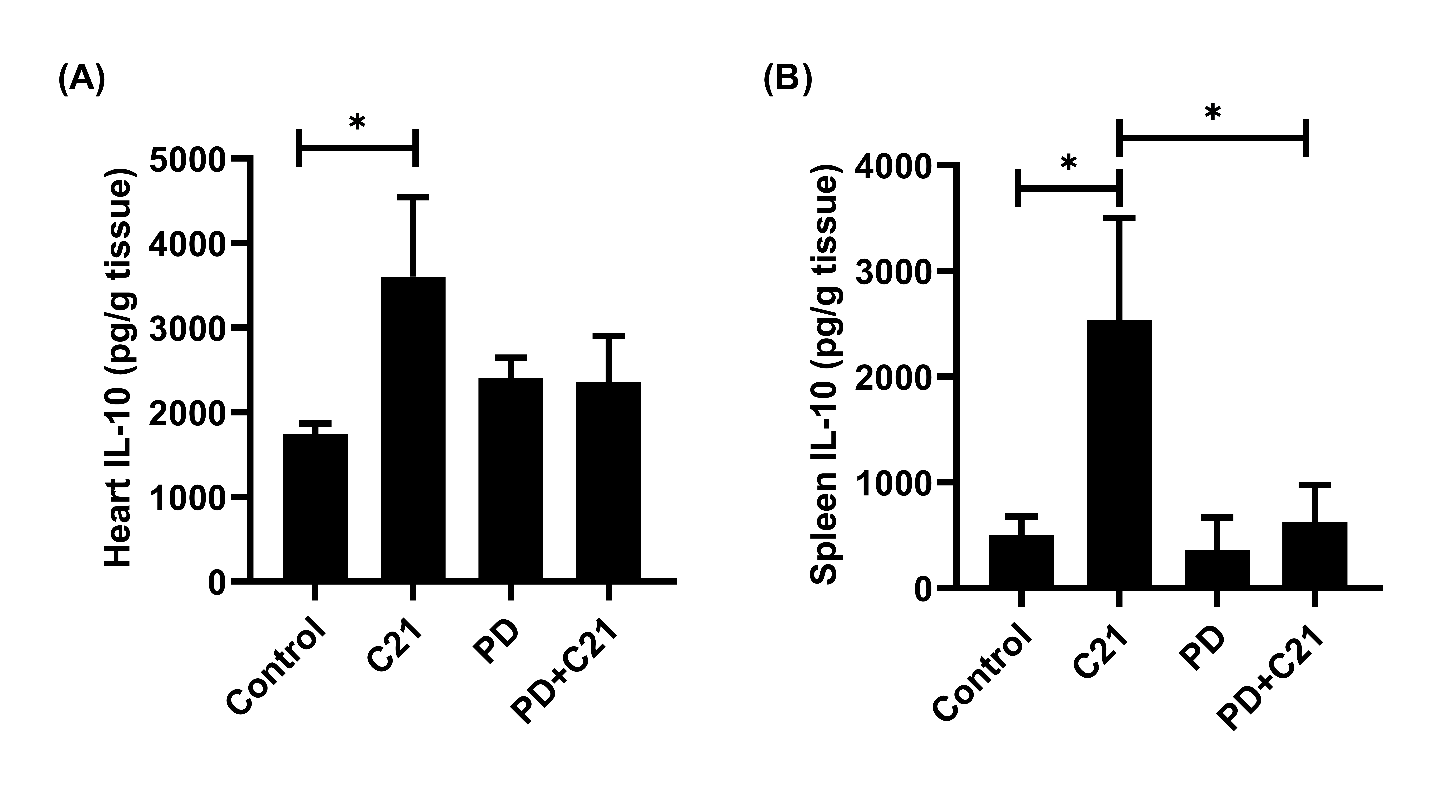
**

**Supplementary Figure 2:** Inhibition of anti-inflammatory effect of C21 by AT2R antagonist PD in the **(A)** heart and **(B)** spleen of C57BL6/NHsd mice. Data are represented as Mean ± S.E.M., analyzed by one-way ANOVA with Fisher’s LSD test for multiple comparisons and are considered significant at **P*<0.05.


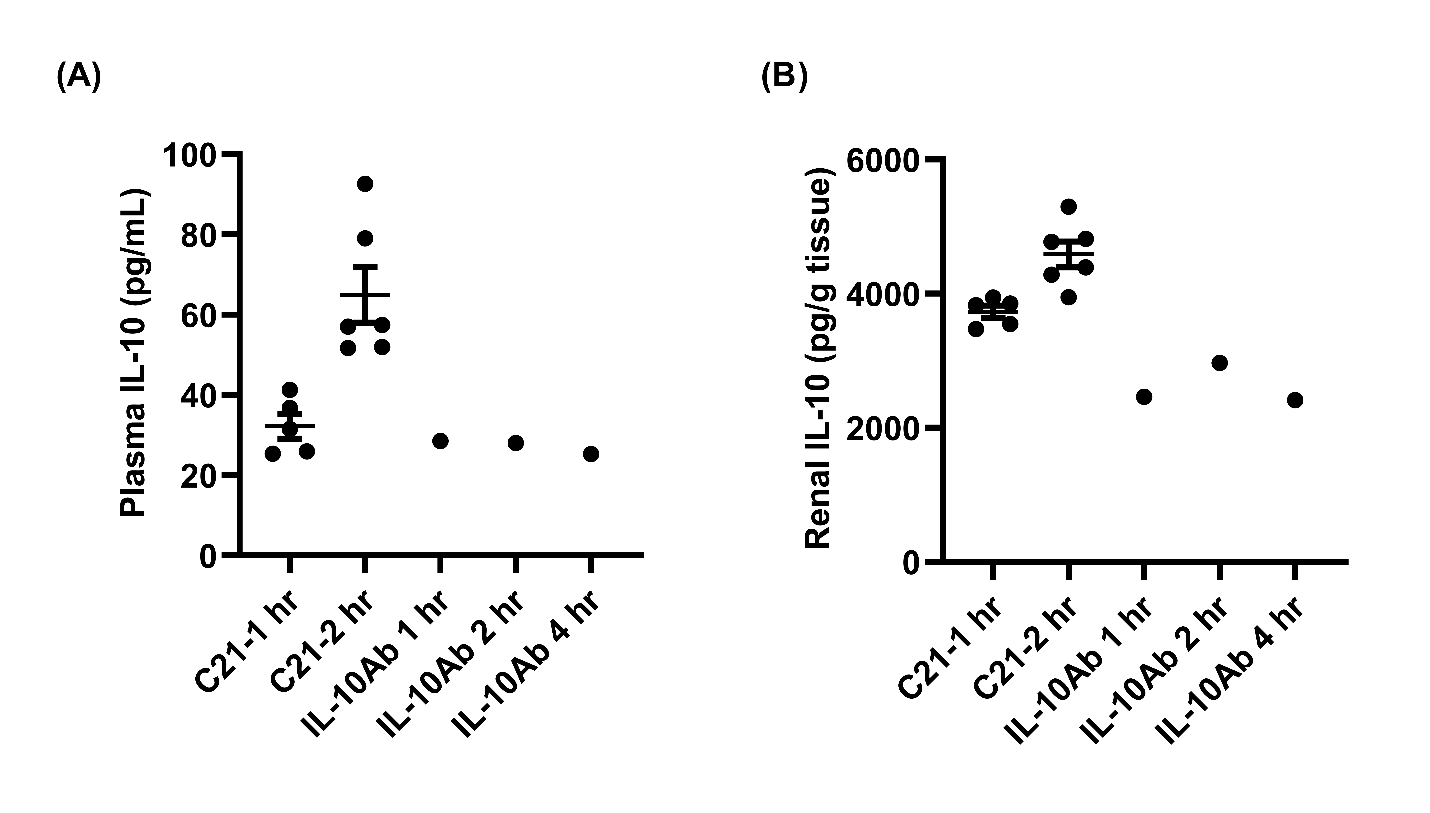


**Supplementary Figure 3:** Reduction in free IL-10 levels in the **(A)** plasma and **(B)** kidney of C57BL6/NHsd mice by treatment with neutralizing IL-10 antibody at a dose of 200 μg/mice.
